# Supplementary material for: High-affinity tuning of single fluorescent protein-type indicators by flexible linker length optimization in topology mutant
Source: Commun Biol. 2024 Jun 8;7:705. doi: 10.1038/s42003-024-06394-0 (PMC11162441; doi:10.1038/s42003-024-06394-0)
Supplement: Supplementary file 2 — Supplementary Information [file 42003_2024_6394_MOESM2_ESM.pdf]

Supplementary Information for:

High-affinity tuning of single fluorescent protein-type indicators by flexible linker length optimization in topology mutant

Yusuke Hara, Aya Ichiraku, Tomoki Matsuda, Ayuko Sakane, Takuya Sasaki, Takeharu Nagai, and Kazuki Horikawa

-Supplementary Figures 1–12

-Supplementary Tables 1–2

-Supplementary Note 1–2

-Supplementary Movies 1–3

estimated resting $[Ca^{2+}]_{in}$ (nM)	organism	celltype	GECI	GECI's K_d (nM)	references
10-15	*D. discoideum*	developing cell	YC-Nano15	15	Ref(6)
			Campari-nano	19	This work
23-37	*P. falciparum*	trophozoite stage	YC-nano50	48.5	Ref(3)
32-59	*R. norvegicus*	pyramidal neuron	OGB1	206	Ref(17)
40-50	*A. thaliana*	root cell	YC-Nano65	65	Ref(4)
40-90	*R. norvegicus*	astro-glia	OGB2 (FLIM)	158	Ref(16)
	M.musculus	astro-glia	YC-Nano50	48.5	Ref(5)

Supplementary Fig. 1. Known resting $[Ca^{2+}]_{in}$ at the low nM range.
Representative of resting $[Ca^{2+}]_{in}$ at low nM in various celltypes and organisms. Sorted by resting $[Ca^{2+}]_{in}$.

a

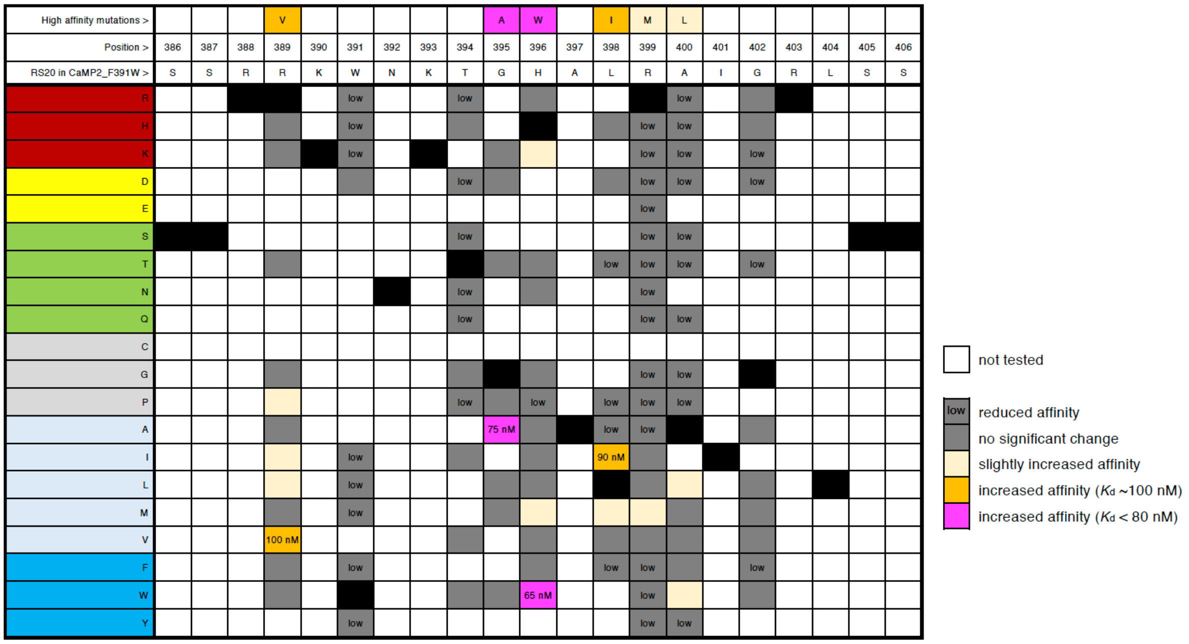

b

mutations on RS20	K_d (approx.)
L398	121 nM
R389V	100 nM
L398I	90 nM
G395A	75 nM
G395A, R389V	75 nM
G395A, A400L	75 nM
G395A, L398I	70 nM
G395A, H396W	60 nM
H396W	65 nM
H396W, L391I	55 nM
G395A, H396W, L398I	45 nM

Supplementary Fig. 2. High-affinity mutations in RS20 of CaMP2_F391W.
Screening of high-affinity mutations in RS20 of CaMP2_F391W. Gray represents tested mutations yielding preserved- or lower affinity (low). Colors discriminate the magnitude of increased affinity, where magenta shows the higher affinity. (**b**) Effects of combinations of selected mutations in (a). We first focused on G395A since it is a known mutation increasing affinity in CaMPARI2[10]. The combination of G395A with R389V or A400L did not increase Ca^{2+} affinity, and R399M was found to reduce the dynamic range. On the other hand, L398I and H396W were found to increase Ca^{2+} affinity when combined with G395A. We finally tested the triple mutation and found that G395A, H396W, and L398I further increased Ca^{2+} affinity than those in any of the two without affecting other properties, such as dynamic range, brightness, and photoconvertibility.

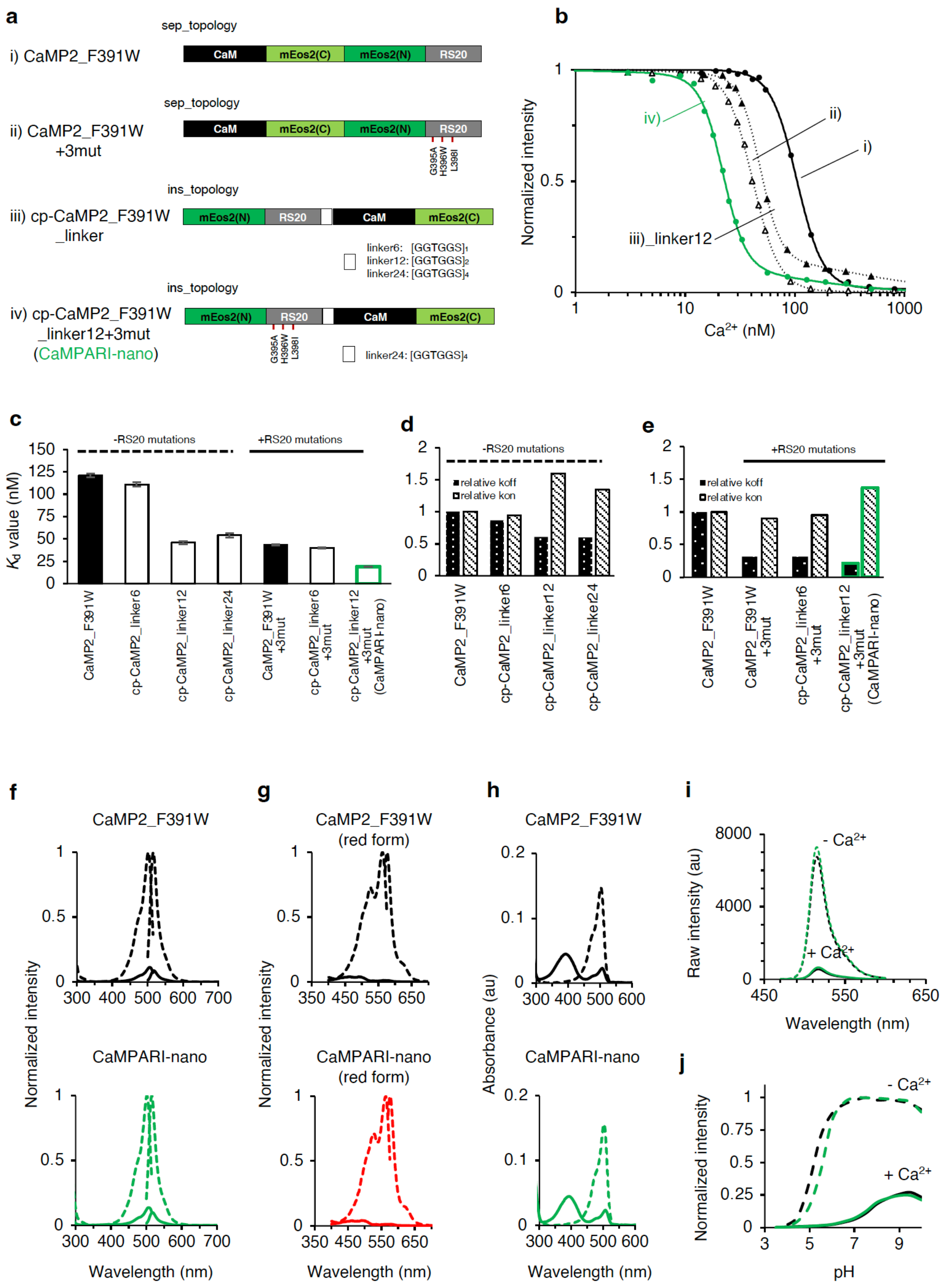

Supplementary Fig. 3. Design and properties of CaMPARI-nano.

(a, b) Molecular design (a) and Ca^{2+} titration (b) of the parental CaMPARI2_F391W (CaMP2_F391W) (i), CaMP2_F391W introduced with three high-affinity mutations in RS20 (ii), circularly permuted CaMP2_F391W (iii), and circularly permuted CaMP2_F391W harboring RS20-mutations (iv). (**c**) Effects of the linker length variation on Ca^{2+} affinity in the absence (dashed) or presence (solid) of RS20-mutations. The filled and open box discriminates separation and insertion topology. Estimated K_d value was plotted with uncertainty (error bars) in Hill fitting of the averaged trace from three independent measurements. (**d, e**) Relative change of k_{on} and k_{off} values compared to that of parental CaMP2_F391W in the absence (d) or presence (e) of RS20-mutations. (**f–j**) Comparative measurement of the excitation and emission intensities (f, g), absorbance (h), brightness (i), and (pH sensitivity) for equimolar CaMPARI-nano (green or red) and parental CaMP2_F391W (black) in the presence (solid lines) or absence (dashed lines) of Ca^{2+}.

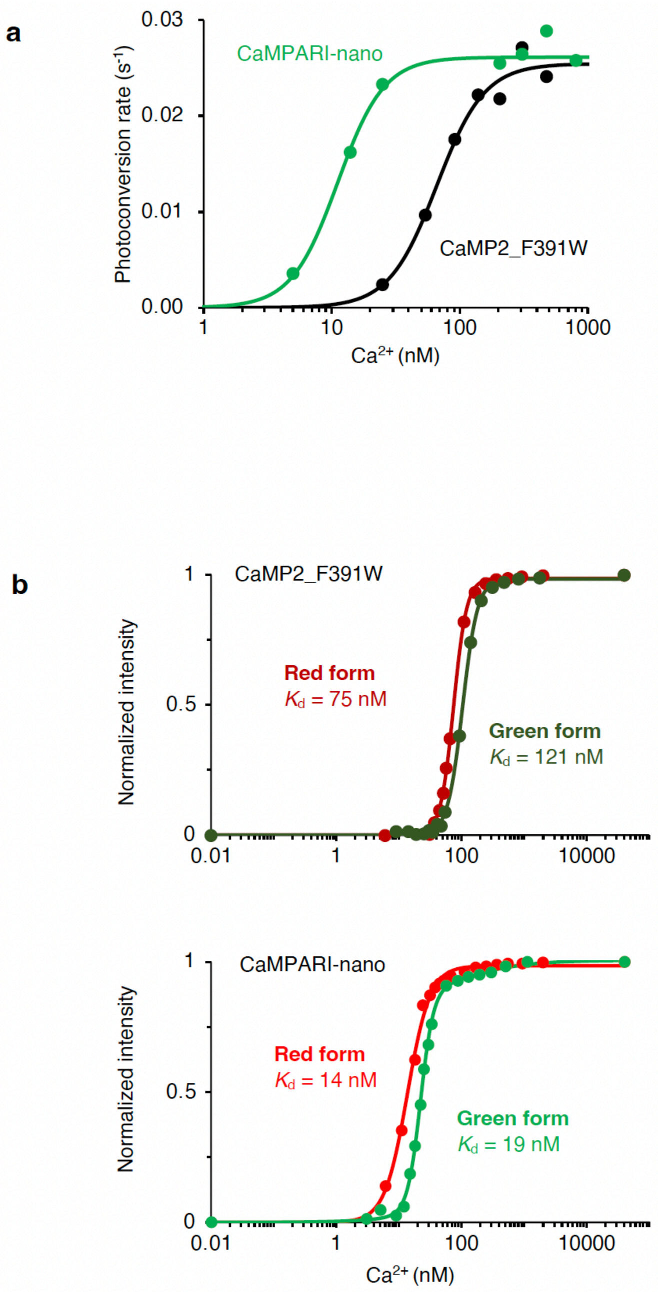

Supplementary Fig. 4. Photoconversion properties of parental and developed CaMPARIs. **(a)** Photoconversion rate constants titrated using different Ca^{2+} concentrations. **(b)** Ca^{2+} titration of the green and red forms of CaMPARIs.

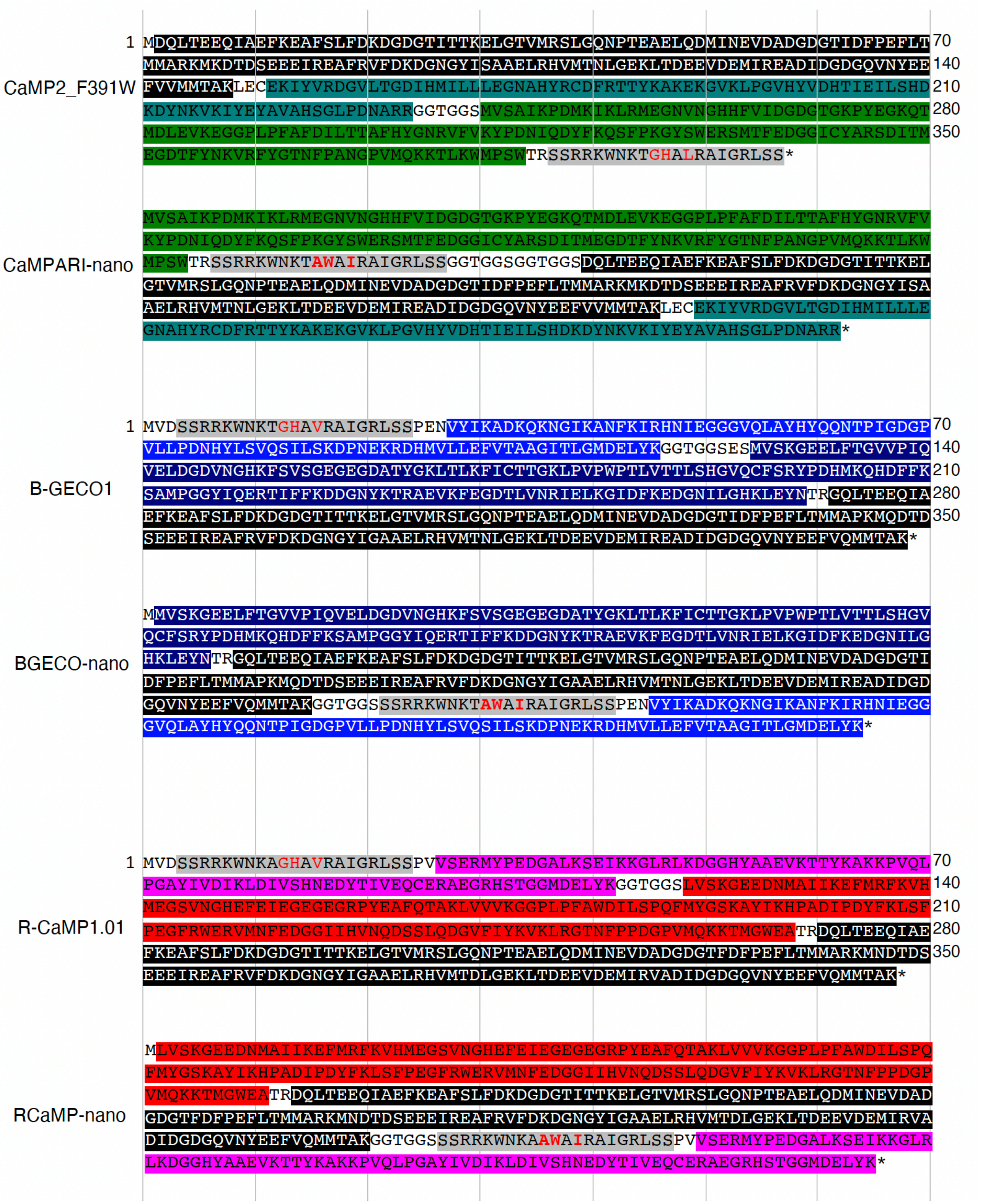

Supplementary Fig. 5. Primary sequences of parental and developed GECIs.
Gray and black represent RS20 and CaM moieties, respectively. Red characters indicate mutated amino acids in the high-affinity GECIs. Note that RCaMP-nano harbors an additional mutation in RS20 (T394A) derived from parental R-CaMP1.01.

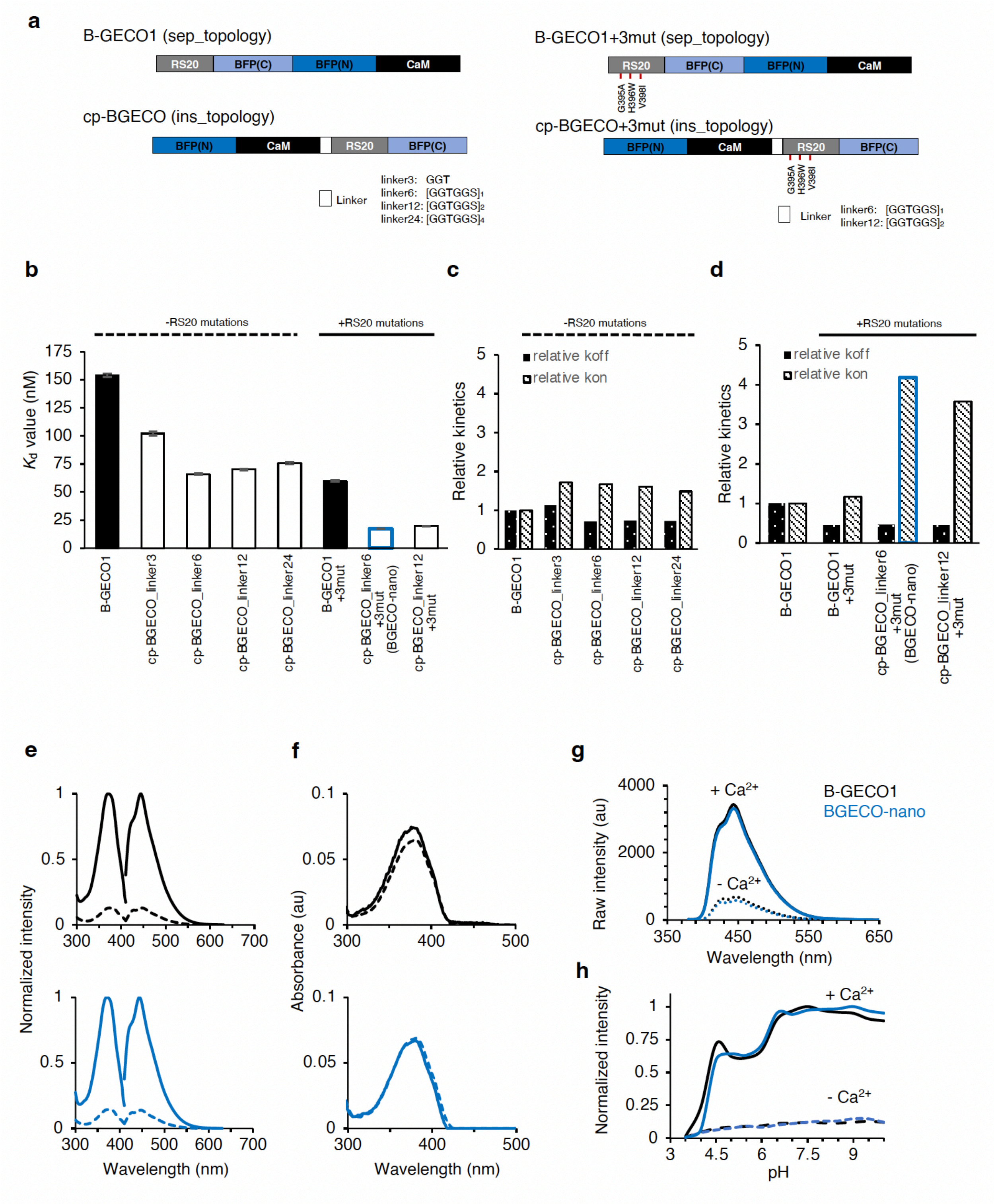

Supplementary Fig. 6. Design and properties of BGECO-nano

(a) Molecular design of the parental B-GECO1 (sensor separated topology) and variants of cp-BGECO for linker length (insertion topology) without (left) or with (right) RS20-mutations. (**b**) Effects of the linker length variation on Ca^{2+} affinity in the absence (dashed) or presence (solid) of RS20-mutations. The filled and open box discriminates separation and insertion topology.

Estimated K_d value was plotted with uncertainty (error bars) in Hill fitting of the averaged trace from three independent measurements. (**c, d**) Relative change of k_{on} and k_{off} values compared to that of parental B-GECO1 without (c) or with (d) RS20-mutations. (**e–i**) Comparative measurement of the excitation and emission intensities (e), absorbance (f), brightness (g), and pH sensitivity (h) for equimolar BGECO-nano (green or red) and parental B-GECO1 (black) in the presence (solid lines) or absence (dashed lines) of Ca^{2+}.

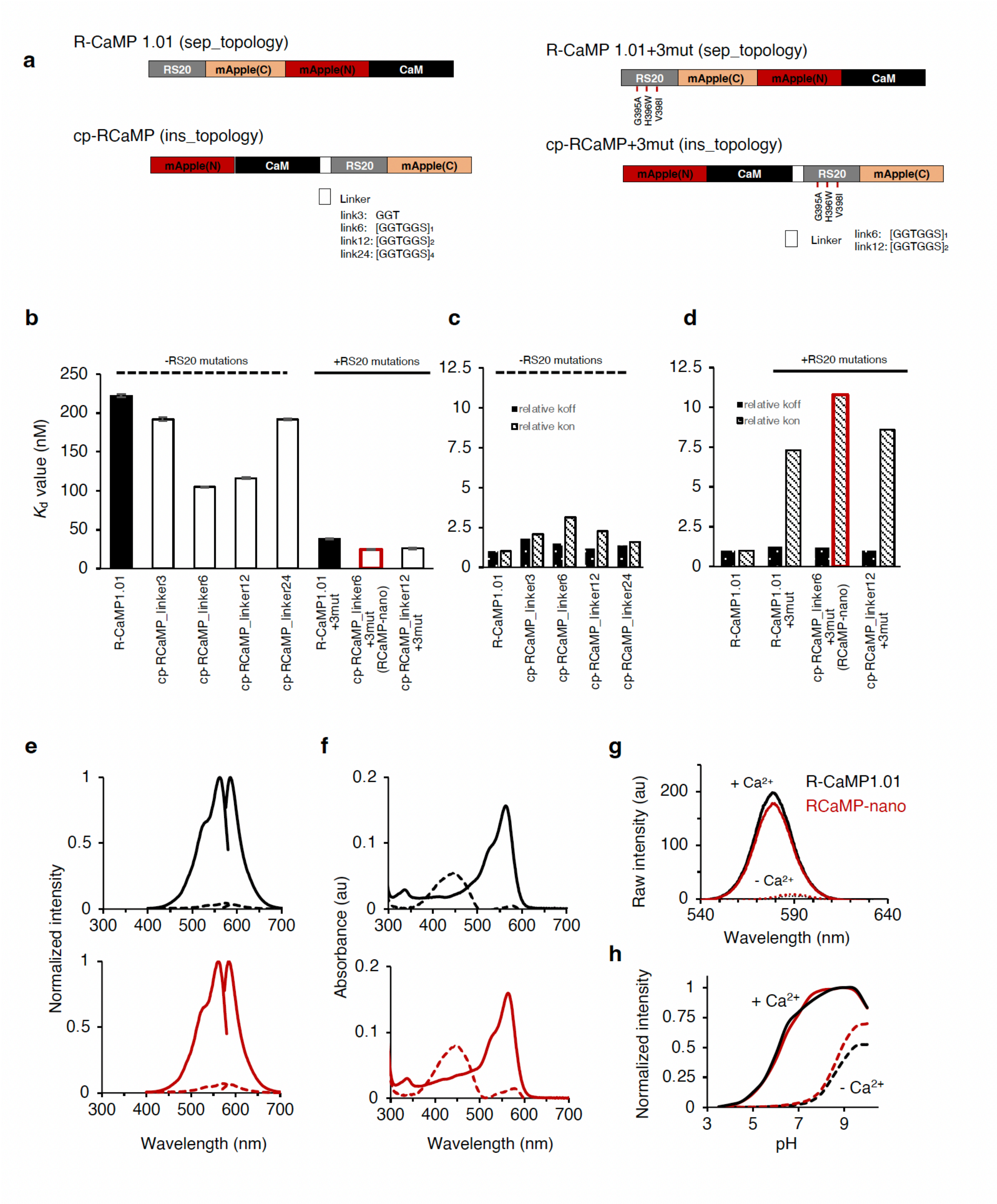

Supplementary Fig. 7. Design and properties of RCaMP-nano

(a) Molecular design of the parental R-CaMP1.01 (sensor separated topology) and variants of cp-RCaMP for linker length (insertion topology) without (left) or with (right) RS20-mutations. **(b)** Effects of the linker length variation on Ca^{2+} affinity in the absence (dashed) or presence (solid) of RS20-mutations. The filled and open box discriminates separation and insertion topology.

Estimated K_d value was plotted with uncertainty (error bars) in Hill fitting of the averaged trace from three independent measurements. (**c, d**) Relative change of k_{on} and k_{off} values compared to that of parental R-CaMP1.01 without (c) or with (d) RS20-mutations. (**e–i**) Comparative measurement of the excitation and emission intensities (e), absorbance (f), brightness (g), and pH sensitivity (h) for equimolar RCaMP-nano (green or red) and parental R-CaMP1.01 (black) in the presence (solid lines) or absence (dashed lines) of Ca^{2+}.

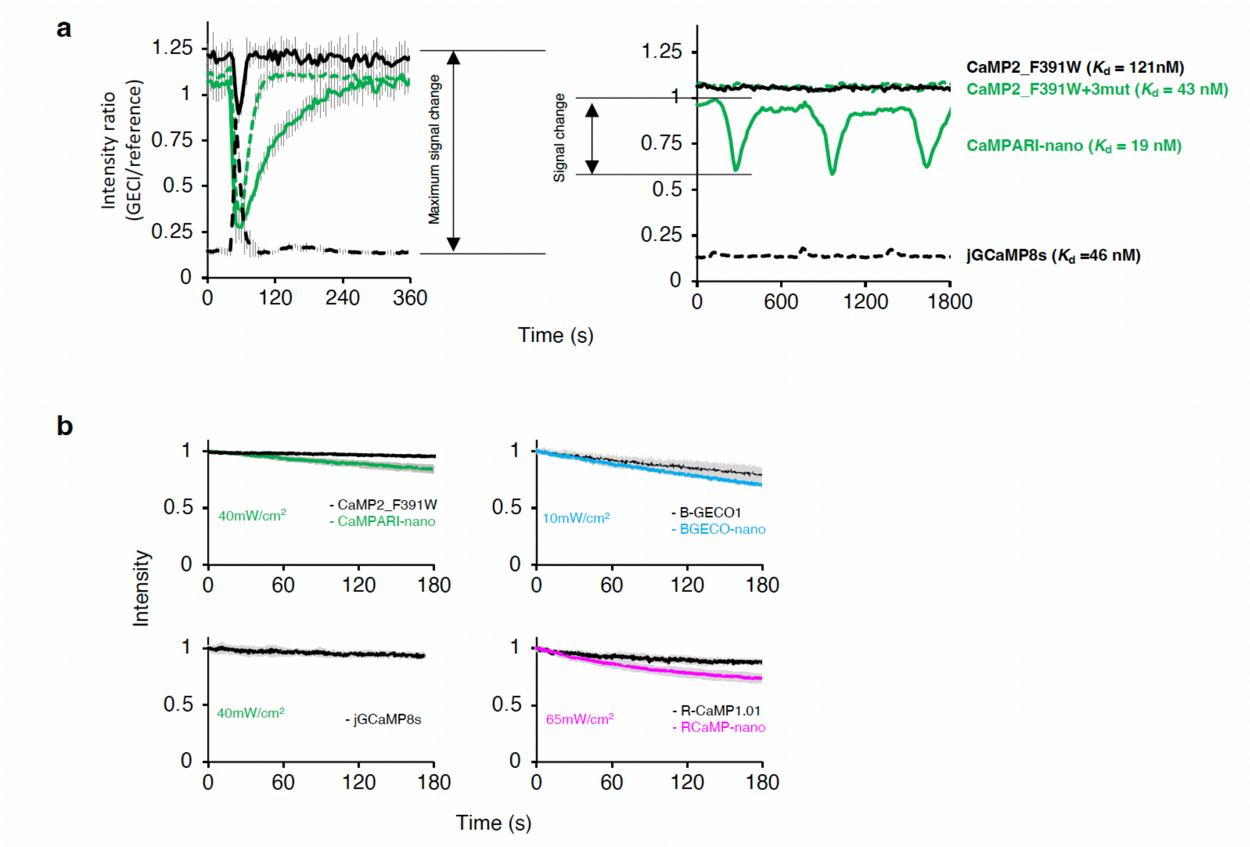

Supplementary Fig. 8. *in-cell* performance of developed GECIs.
(a) Performance of GECIs with moderately high affinity having K_d ~45 nM in the detection of induced (left) and spontaneous $[Ca^{2+}]_{in}$ transients (right) in *D. discoideum cells*. The magnitude of signal change in Fig. 2c was calculated from "signal change (left)" divided by "maximum signal change (right)". To calculate the maximum signal change in *Dictyostelium discoideum* cells where BAPTA-AM and ionomycin treatment are not functional, R_{min} and R_{max} for each GECI group were determined as follows. For the green GECI group, the resting intensity ratio of flashing type jGCaMP8s was considered as $R_{min,}$ and the resting intensity ratio of inversely flashing type CaMP2_F391W was considered as R_{max}. For the blue and red GECI group, R_{min} and R_{max} were obtained by the intensity ratio of low-affinity parental GECIs at resting and that of ultrahigh-affinity GECIs at the peak response upon forced cAMP stimulation, respectively. Mean±s.e.m. of five cells (left) and representative data in single ROI containing ~10 cells (left). (**b**) Photobleaching time course under continuous illumination with shown power densities *in D. discoideum cells*. Mean±s.e.m.. Shown are the data in a representative single experiment whose reproducibility was confirmed by three and two independent experiments for (a) and (b), respectively.

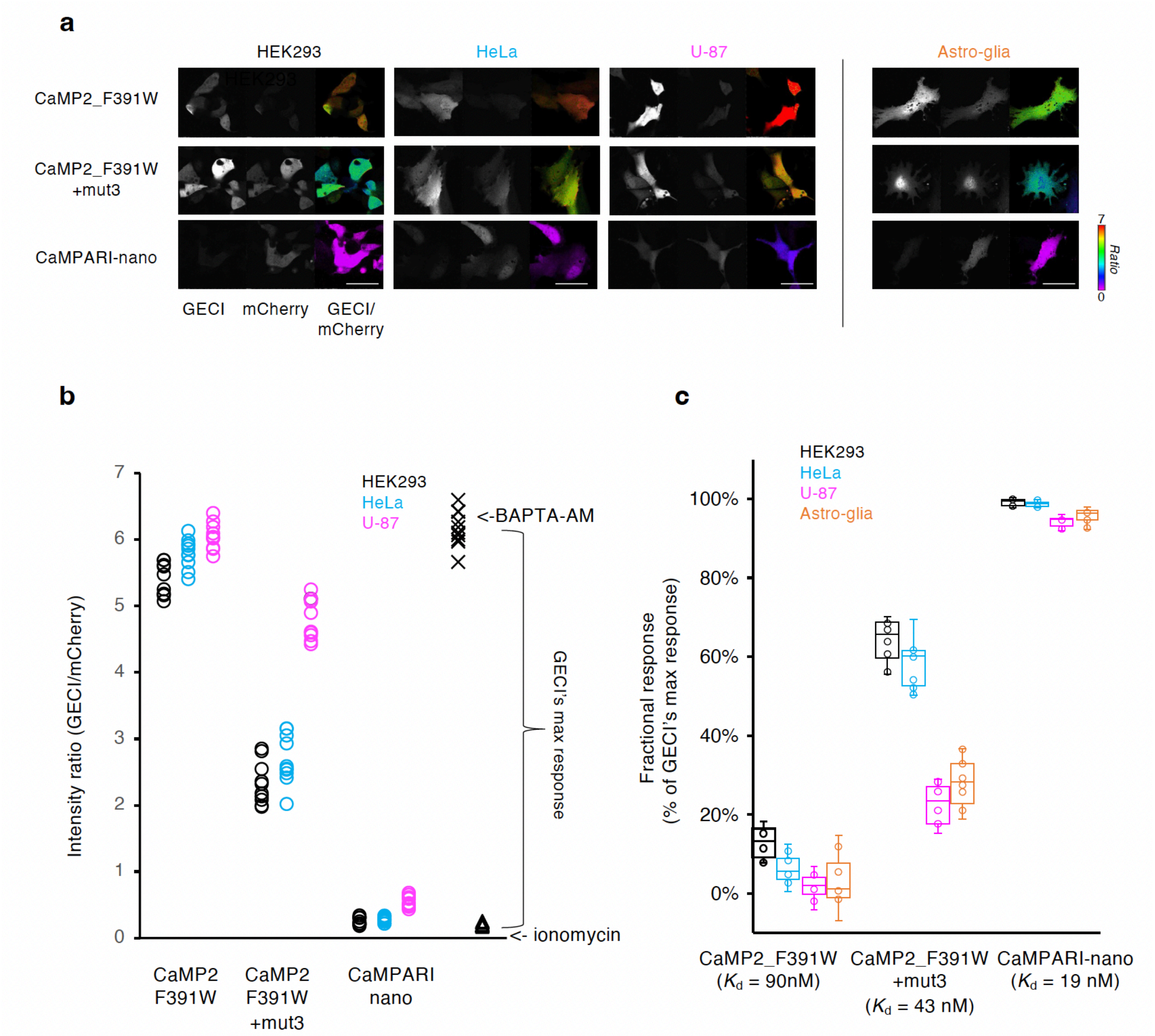

Supplementary Fig. 9. Estimation of resting $[Ca^{2+}]_{in}$ among diverse celltypes.

(a) The cytoplasmic expression of GECIs, reference mCherry, and ratio image of GECI/reference in HEK293, HeLa, U-87 cells at 48 h after the transfection. Those of astro-glia at 4 days after transfection. Scale bar: 0.05 mm. (**b**) Resting $[Ca^{2+}]_{in}$ detected by affinity variants of CaMPARIs. The intensity ratio in resting HEK293, HeLa, and U-87 cells (N = 10 cells) was shown. The range of GECI's maximum response was determined by the intensity ratio in BAPTA-AM or ionomycin-treated cells. (**c**) Boxplot of the fractional changes of each GECIs. The range of GECI's maximum response in astro-glia (N = 9 cells) was analyzed by using independently determined R_{max} (4.1) and R_{min} (0.1), since its imaging condition (4 days after transfection) was different from the other three celltypes (48 h after transfection). All data points, median, interquartile points, and 1.5× interquartile range were shown, respectively. Shown are the data in a representative single experiment whose reproducibility was confirmed by three independent experiments.

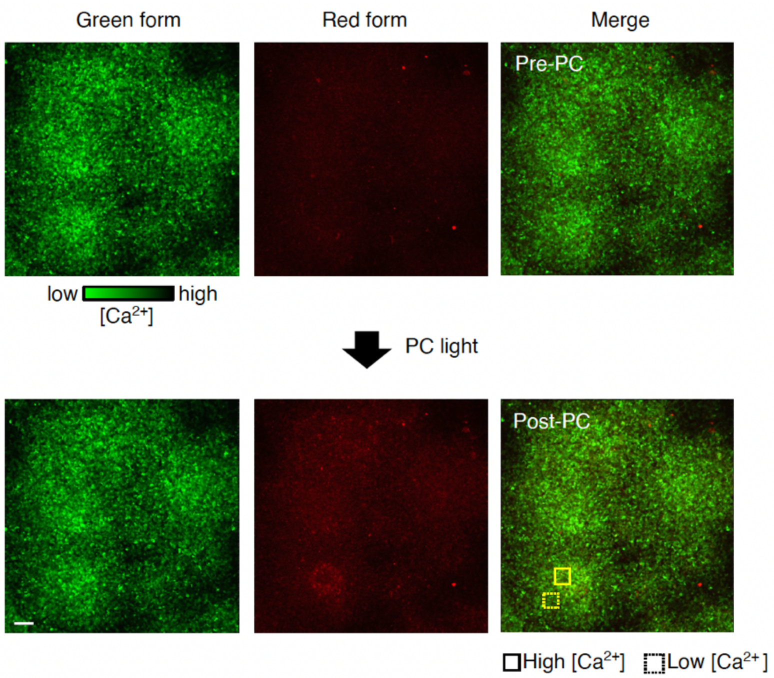

Supplementary Fig. 10. Unsuccessful photoconversion of cells expressing a low-affinity CaMPARI.
Green, red, and merged channels before and after the PC light illumination on cells expressing parental CaMP2_F391W.

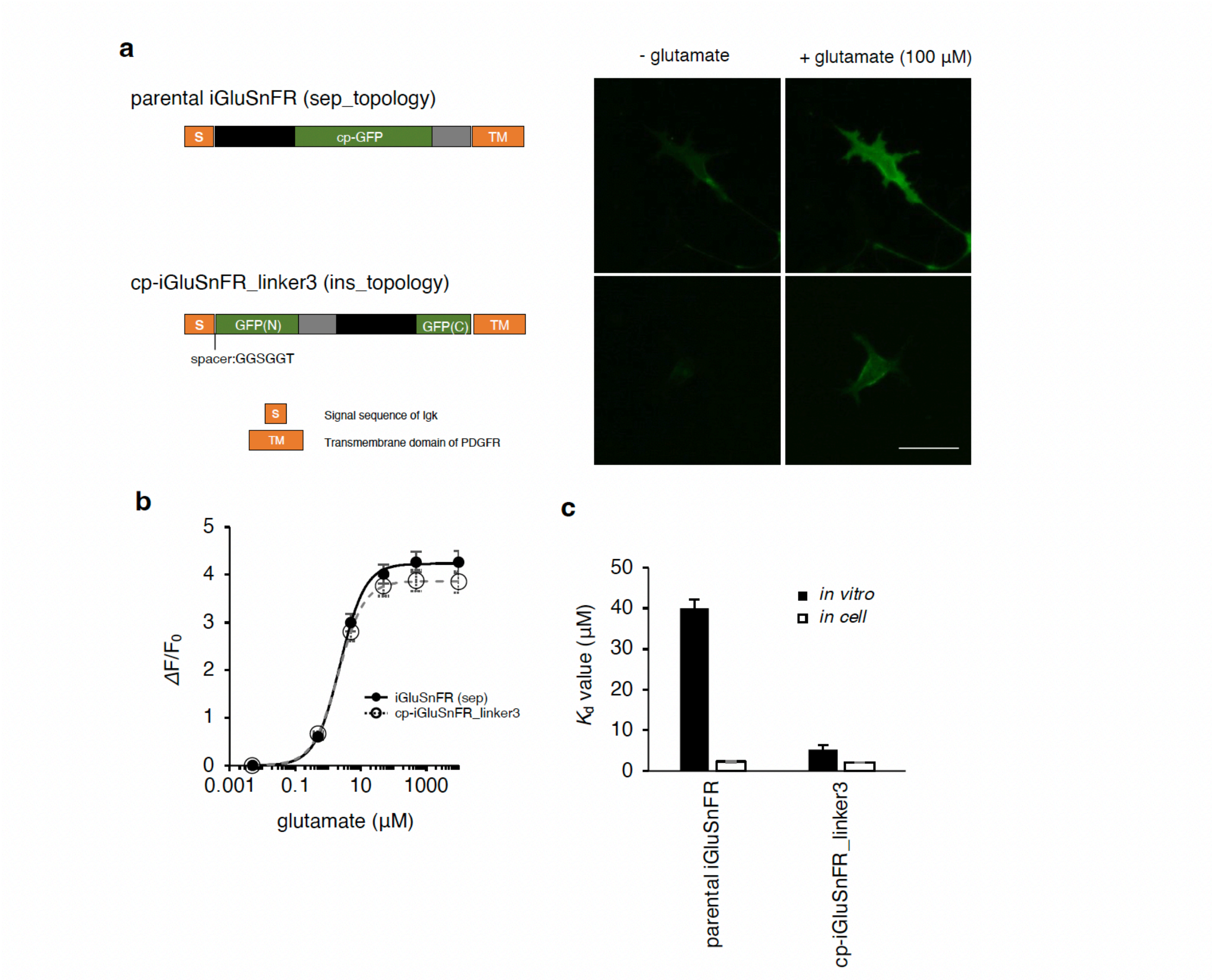

Supplementary Fig. 11. *in-cell* performance of iGluSnFR variant.
(**a**) Cell surface expression of parental iGluSnFR (top) and cp-iGluSnFR (top) in HEK293 cells at 48 h after the transfection.. A spacer was added for efficient processing by a signal peptidase. Scale bar: 0.05 mm. (**b**) Titration of surface displayed iGluSnFRs. (**c**) K_ds of purified (filled) and cell-surface exposed (open) indicators. Parental iGluSnFR showed significant increase of *in-cell* affinity (2.3 μM) as reported previously[31, 33], while cp-iGluSnFR did not (2.1 μM). Estimated K_d value was plotted with uncertainty (error bars) in Hill fitting of the averaged trace from three independent measurements.

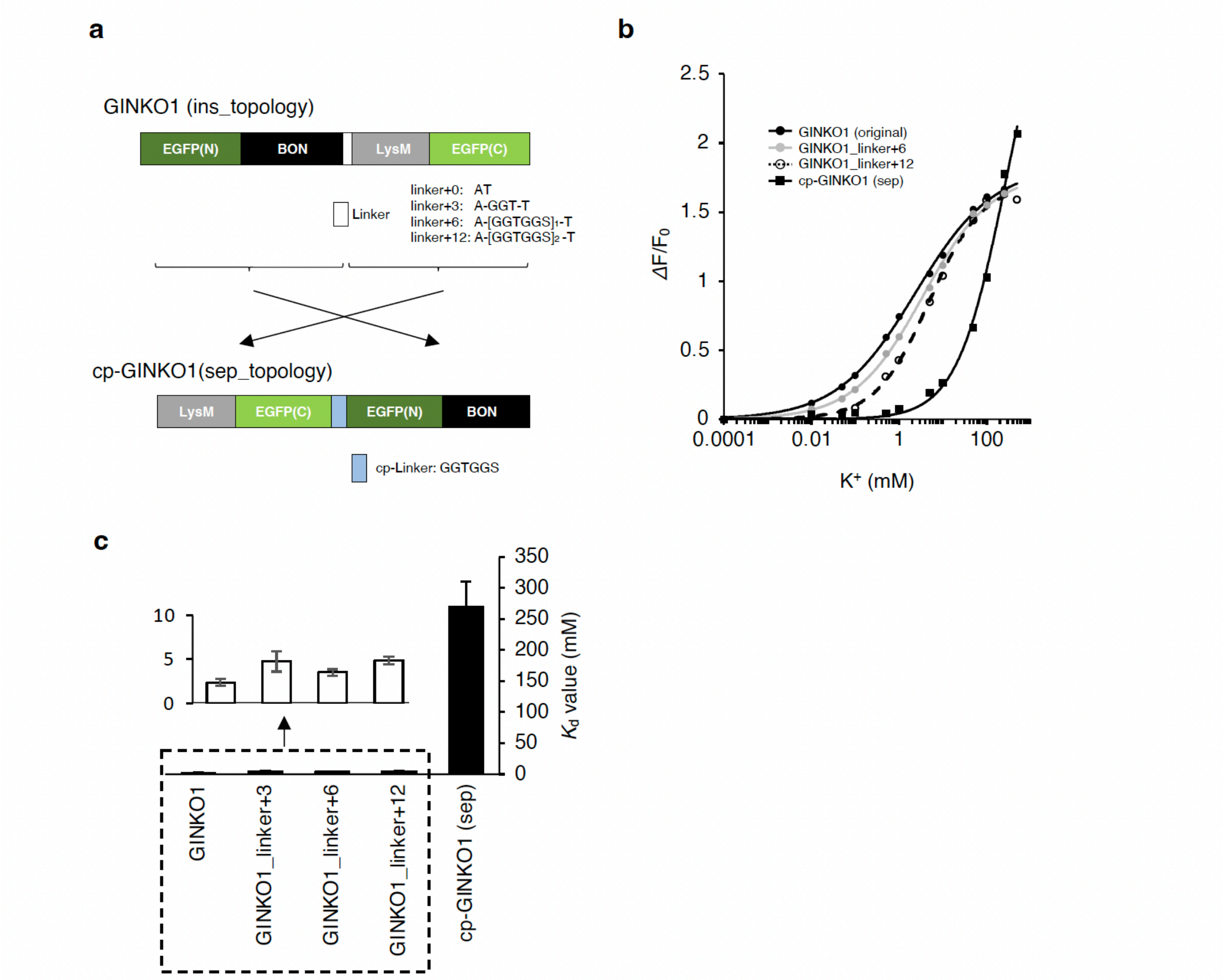

Supplementary Fig. 12. Effects of cp-linker and the topology mutant in K^+ indicator, GINKO1.

(a) Linker length variation in GINKO1 originally having an insertion topology (top). Circularly permuted GINKO1 having a sensor separated topology (bottom). (**b**) Titration curve for purified proteins of modified GINKOs. (c) K_d values of modified GINKOs. Estimated K_d value was plotted with uncertainty (error bars) in Hill fitting of the averaged trace from three independent measurements.

Supplementary Table 1. Properties of parental and developed GECIs.

Indicator	Ca^{2+}	λ_{ex}	λ_{em}	relative brightness*	pK_a	$\Delta F/F_0$	K_d (nM)	Hill	k_{on} (10^6, $M^{-1}s^{-1}$	k_{off} (s^{-1})
CaMP2_F391W (green state)	-	502	515	1	5.1	10.7	121	3.4	76.5	0.93
	+	505	517	0.085	7.6					
cp-CaMP2_F391W_linker6	-	502	516	*n.d.*	5.2	10.8	111	3.5	72.1	0.80
	+	505	517	*n.d.*	7.5					
cp-CaMP2_F391W_linker12	-	502	515	*n.d.*	5.2	10.1	46	3.3	122.1	0.56
	+	505	517	*n.d.*	7.4					
cp-CaMP2_F391W_linker24	-	502	515	*n.d.*	5.3	11	54	3.4	102.9	0.56
	+	505	517	*n.d.*	7.4					
CaMP2_F391W +3mut	-	502	516	*n.d.*	5.2	13.2	43	3.3	68.6	0.29
	+	505	517	*n.d.*	7.7					
cp-CaMP2_F391W_linker6 +3mut	-	502	515	*n.d.*	5.2	11.5	40	3	72.9	0.29
	+	505	517	*n.d.*	7.5					
cp-CaMP2_F391W_linker12 +3mut **(CaMPARI-nano)**	-	502	515	1.15	5.7	11.6	19	3.2	104.6	0.20
	+	505	517	0.1	7.4					
jGCaMP8s	-	400	516	0.015	*n.d*	47.5	46	2.2	77.6	3.57
	+	495	513	0.88	*n.d*					

indicator	Ca^{2+}	λ_{ex}	λ_{em}	relative brightness*	pK_a	$\Delta F/F_0$	K_d (nM)	Hill	k_{on} (10^6, $M^{-1}s^{-1)}$	k_{off} (s^{-1})
B-GECO1	-	373	447	0.2	4.4	5.2	154	3.3	76.4	1.17
	+	371	445	1	5					
cp-BGECO_linker3	-	373	445	*n.d.*	5.9	2.6	102	2.9	130.7	1.33
	+	372	443	*n.d.*	4.8					
cp-BGECO_linker6	-	372	445	*n.d.*	4.5	3.8	66	2.5	127.3	0.84
	+	372	443	*n.d.*	4.6					
cp-BGECO_linker12	-	372	445	*n.d.*	5.2	3.5	70	2.8	123.2	0.86
	+	372	444	*n.d.*	4.9					
cp-BGECO_linker24	-	371	447	*n.d.*	4.9	3.4	75.6	3.2	113.1	0.85
	+	370	445	*n.d.*	4.8					
B-GECO1 +3mut	-	375	446	*n.d.*	4.3	5.7	59.8	2.9	89.4	0.53
	+	371	444	*n.d.*	4.4					
cp-BGECO_linker6 +3mut **(BGECO-nano)**	-	372	446	0.17	4.9	4.3	17.3	2.3	319.4	0.55
	+	371	444	0.92	4.7					
cp-BGECO_linker12 +3mut	-	373	445	0.16	5.1	5	19.6	2.1	272.8	0.53
	+	371	443	0.92	4.7					

indicator	Ca^{2+}	λ_{ex}	λ_{em}	relative brightness*	pK_a	$\Delta F/F_0$†	K_d (nM)	Hill	k_{on} (10^6, $M^{-1}s^{-1)}$	k_{off} (s^{-1})
R-CaMP1.01	-	574	590	0.05	8.6	28.7	222	1.8	336.2	7.46
	+	561	579	1	6.2					
cp-RCaMP_linker3	-	575	589	*n.d.*	8.6	19.6	192	1.9	694.4	13.3
	+	562	578	*n.d.*	6.3					
cp-RCaMP_linker6	-	575	589	*n.d.*	8.6	23.6	105	1.8	1046.6	10.99
	+	561	578	*n.d.*	6.1					
cp-RCaMP_linker12	-	574	589	*n.d.*	8.7	22.9	116	1.8	762.9	8.85
	+	561	578	*n.d.*	6.2					
cp-RCaMP_linker24	-	576	589	*n.d.*	8.6	20.3	192	1.8	536.9	10.31
	+	561	578	*n.d.*	6.1					
R-CaMP1.01 +3mut	-	574	588	*n.d.*	8.6	25.8	37.8	1.6	2449.5	9.26
	+	561	578	*n.d.*	6.3					
cp-RCaMP_linker6 +3mut **(RCaMP-nano)**	-	574	587	0.076	8.6	16.5	24.4	1.3	3626.9	8.85
	+	562	578	0.92	6.1					
cp-RCaMP_linker12 +3mut	-	573	587	0.076	8.6	16.2	25.6	1.4	2893.5	7.41
	+	561	578	0.92	6.2					

* Relative brightness to F_{max} of the parental indicator under equimolar measurement.

† To calculate the fold change of red-color indicators showing ~10 nm blue shift upon Ca^{2+}-binding, the intensity at 589 nm is used for both (+) and (-) Ca^{2+}-conditions, as reported previously[13].

n.d., not determined.

Supplementary Table 2. Properties of parental and developed indicator for glutamate and K^+.

indicator	glutamate	λ_{ex}	λ_{em}	relative brightness*	pKa	$\Delta F/F_0$	K_d (μM)	Hill co.
iGluSnFR (sep)	-	495	512	0.19	7.7	5.3	40 ±0.81	1
	+	493	510	1	6.1			
cp-iGluSnFR_linker3	-	496	512	0.16	7	7.3	6.2 ±0.17	1.1
	+	493	511	1.19	6			
cp-iGluSnFR_linker6	-	495	511	0.2	7	5.1	7 ±0.43	0.9
	+	493	510	1.1	6			
cp-iGluSnFR_linker12	-	495	511	0.19	7	5.9	34 ±0.51	0.9
	+	494	510	1.17	6			
cp-iGluSnFR_linker24	-	495	511	0.23	7	5.5	36 ±0.75	1
	+	493	510	1.15	6			

indicator	K+	λ_{ex}	λ_{em}	relative brightness*	pKa	$\Delta F/F_0$	K_d (mM)	Hill co.
GINKO1	-	399	514	0.37	7.7	1.6	2.38 ±0.4	0.48
	+	400	515	1	7.6			
GINKO1_linker+3	-	399	514	0.37	7.7	1.6	4.75 ±1.15	0.47
	+	400	515	1	7.6			
GINKO1_linker+6	-	399	514	0.37	7.7	1.6	3.5 ±0.36	0.55
	+	400	515	1	7.6			
GINKO1_linker+12	-	399	514	0.38	7.7	1.6	4.87 ±0.45	0.71
	+	400	515	1	7.6			
cp-GINKO1 (sep)	-	399	514	0.2	8.4	2.1	270 ±40	0.78
	+	400	515	0.64	8			

* Relative brightness to F_{max} of the parental indicator under equimolar measurement.

Supplementary Note 1

Kinetic effects of the linker length optimization and RS20-mutations

To specify the mechanisms of how linker length optimization and RS20-mutations increased Ca^{2+} affinity, we analyzed k_{off} and k_{on} for all variants of GECIs (**Supplementary Table 1**) and quantified relative changes of these kinetics to parental ones (**Supplementary Fig. 3d,e, 4c,d, 5c,d**). To our surprise, the results revealed linker length optimization and RS20-mutations differently affected on- and off-kinetics in three GECIs as discussed below:

We start with RCaMPs as the most simple case. **Supplementary Fig. 7c** shows how the linker length variation affected on- and off-kinetics in the absence of RS20-mutations. We observed bell shaped increase of k_{on} values peaked at 6 a.a. linker, while k_{off} values were kept almost constant. Such a quite similar result to YC-nano[6] indicated that the linker length optimization specifically accelerated the on-kinetics in RCaMPs. The effect of RS20-mutations on parental R-CaMP1.01 was presented in the first two columns in **Supplementary Fig. 7d**, showing that RS20-mutations also accelerated the on-kinetics at a larger contribution (×7.5) than linker length optimization (×2.5, **Supplementary Fig. 7c**). The combination of optimized cp-linker (6 a.a.) and RS20-mutations further accelerated on-kinetics without affecting off-kinetics (**Supplementary Fig. 7d**, red). Fold change in the increase of k_{on} was nearly ×10, suggesting that optimized cp-linker (6 a.a.) and RS20-mutations <u>additively</u> accelerated on kinetics.

Different from RCaMPs, the cp-linker length optimization of BGECOs affected both on- and off-kinetics with a 1.7-fold acceleration of on- and slowing down of off-kinetics (×0.7) (**Supplementary Fig. 6c**). RS20-mutations also affected both on- and off-kinetics with a slowing down of off- (×0.5) and acceleration of on-kinetics (×1.2) compared to parental B-GECO1. A combination of cp-linker (6 a.a.) and RS20-mutations yielded no further slowing down of off-kinetics but a significant acceleration of on-kinetics than B-GECO1+3mut. The 4-fold increase of k_{on} was far larger than that of cp-linker (×1.7) or RS20-mutations (×1.2), suggesting that the effect of these two would be <u>cooperative</u>.

Cp-linker optimization and RS20-mutations on CaMP2_F391W also changed both on- and off-kinetics but in a pattern different from that of RCaMPs and BGECOs. **Supplementary Fig. 3d** showed that linker length optimization in topology mutant affected both on- and off-kinetics with a 1.6-fold acceleration of on- and a 0.6-fold slowing down of off-kinetics as observed for BGECOs. RS20-mutations specifically slowed down off-kinetics (×0.3) compared to parental CaMP2_F391W, demonstrating that RS20-mutations affected on- and off-kinetics differently in three tested GECIs. A combination of cp-linker (12 a.a.) and RS20-mutations showed a further slowing down of off-kinetics and acceleration of on-kinetics than that of CaMP2_F391W+3mut. The fold change of k_{on} and k_{off} in CaMPARI-nano was ×1.4 and ×0.3 than that of parental CaMP2_F391W. These results suggested that optimized cp-linker (12 a.a.) and RS20-mutations changed on- (×1.4) and off-kinetics (×0.6) <u>multiplicatively</u>.

In summary, the linker length optimization or RS20-mutations affected on- and off-kinetics differently in three tested GECI families. The combination of these two modifications similarly increased Ca^{2+} affinity, but the behind mechanism seems to be different in each GECI such that the effects on k_{on} and k_{off} were multiplicative, cooperative, and additive, respectively. We have no

clear reasoning for this, but differences in the molecular configuration of sensing modules (RS20-CaM vs CaM-RS20) or in the sterical relationship between FPs and sensing motifs (related to insertion site and linkers between them) would vary the effect of here tested modifications that would be examined in future studies.

Supplementary Note 2

In vitro and in-cell Performance of iNTnC2

We conducted a comparative analysis to examine the possibility that iNTnC2[15], another high-affinity GECI, may outperform CaMPARI-nano. N-terminally poly-histidine-tagged iNTnC2 was expressed in *E.coli* and the purified proteins were subjected to biochemical analysis. Our Ca^{2+} titration revealed the K_d of iNTnC2 to be 41 nM (**Supplementary Note fig. 1a**), a slightly smaller value than the previously reported (49 nM)[15]. The dynamic range ($\Delta F/F_{min}$) of iNTnC2, originally reported to be as high as 30[15], could not be replicated under our experimental conditions. While the maximum brightness of mNeonGreen-based iNTnC2 was found to be 1.15-fold higher than that of mEOS2-based CaMPARIs, its minimum brightness at Ca^{2+}-saturated conditions was not low enough, leading to a significantly reduced dynamic range ($\Delta F/F_{min}$ = 2.28; **Supplementary Note fig. 1b**).

To further check whether the attenuated dynamic range persisted in human and *Dictyostelium* cells, we conducted comparative *in-cell* studies with CaMPARI-nano. In HeLa cells, we evaluated the maximum and minimum brightness of both iNTnC2 and CaMPARI-nano, both of which are inversely flashing-type GECIs. When cells were treated with 1 µM of ionomycin in the presence of 1 mM extracellular Ca^{2+} concentration, the maximum intensity of CaMPARI-nano, normalized by co-expressed mCherry, reached 6.0 (**Supplementary Note fig. 1c)**, in contrast, iNTnC2's was notably lower at 1.4. Furthermore, after treatment with 15 µM BAPTA-AM, the minimum intensities of both CaMPARI-nano and iNTnC2 were similarly low. This led to a significantly narrower *in-cell* dynamic range for iNTnC2 ($\Delta F/F_{min}$ = 5.3) compared to CaMPARI-nano ($\Delta F/F_{min}$ = 22.5).

To further investigate the potential temperature sensitivity of *in-cell* dynamic range of iNTnC2, Ca^{2+} imaging was performed in *Dictyostelium* cells maintained at their optimal culture temperature of 23°C. We focused on fluorescent intensity under resting conditions because ionomycin and BAPTA-AM are not functional in *Dictyostelium* cells. As depicted in **Figure 2a**, both CaMPARI-nano (K_d = 19 nM) and CaMP2_F391W+mut3 (K_d = 43 nM) had similarly high resting intensities when normalized by co-expressed mRFPmars. However, despite iNTnC2's comparable K_d value and *in-vitro* brightness to CaMPARIs, its resting intensity in *Dictyostelium* cells was significantly lower than CaMPARI-nano's (**Supplementary Note fig. 2**). This indicates the potential instability of iNTnC2 in *Dictyostelium* cells, similar to observations in HeLa cells. Moreover, the $[Ca^{2+}]_{in}$ transient induced by cAMP stimulation was barely detectable with iNTnC2, suggesting the *in-cell* dynamic range of iNTnC2 was significantly lower than that of CaMPARI-nano and jGCaMP8s (**Supplementary Note fig. 2**). Importantly, physiological $[Ca^{2+}]_{in}$ transients associated with spontaneously synthesized cAMP remained undetectable with iNTnC2 (**Supplementary Note fig. 3**).

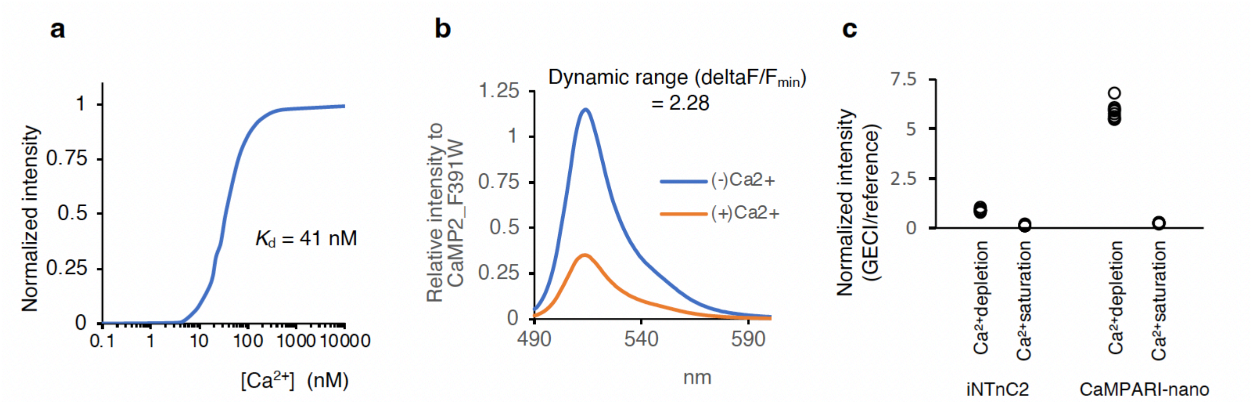

Supplementary Note fig. 1. Properties of iNTnC2. (**a**) Emission spectrum of purified iNTnC2. (**b**) Ca^{2+}-titration curve. (**c**) *in-cell* brightness of iNTnC2. Normalized intensity of HeLa cells expressing iNTnC2_P2AmCherry or CaMPARI-nano_P2AmCherry treated with BAPTA-AM (Ca^{2+}-depletion) or ionomycin (Ca^{2+}-saturation). Representative 10 cells from a single experiment for each condition.

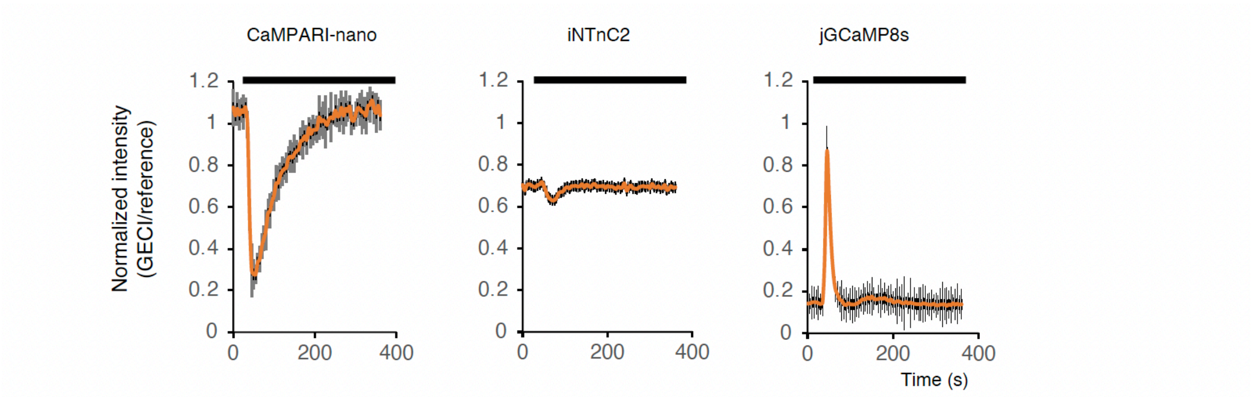

Supplementary Note fig. 2. Performance of iNTnC2 in cAMP stimulated *D.discoideum* cells. Normalized intensity change for CaMPARI-nano, iNTnC2, and jGCaMP8s. Mean±s.e.m.. N = 5 cells. Reproducibility was confirmed by three independent experiments. Bars indicate cAMP stimulation.

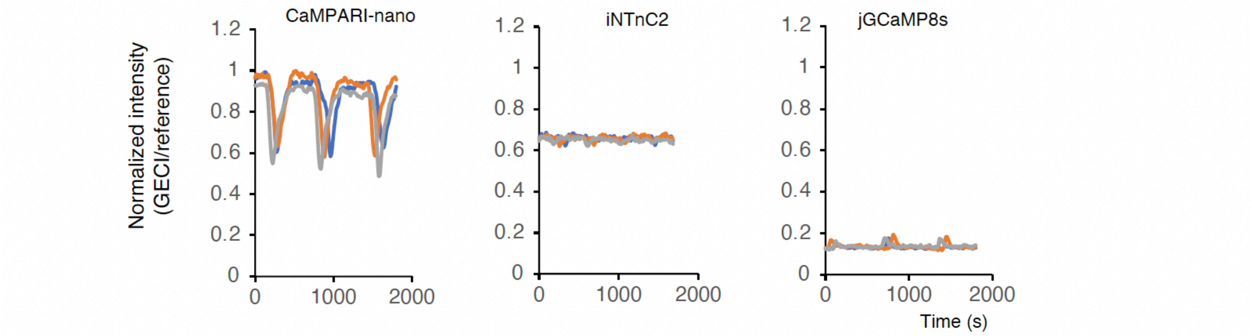

Supplementary Note fig. 3. $[Ca^{2+}]_i$ dynamics for spontaneously signaling *D.discoideum* cells. Normalized intensity of *D.discoideum* cells expressing CaMPARI-nano, iNTnC2, and jGCaMP8s. Traces of three representative ROIs each containing ~10 cells.

Supplementary Movie 1. Comparative [Ca^{2+}] imaging. (AVI, 17.8 MB)

Supplementary Movie 2. Functional highlighting of actively signaling cells by CaMPARI-nano. (AVI. 19.3 MB)

Supplementary Movie 3. Triple-function imaging of Ca^{2+}, cGMP, and cAMP. (AVI. 4.1 MB)
