# Supplementary material for: High-affinity tuning of single fluorescent protein-type indicators by flexible linker length optimization in topology mutant
Source: Commun Biol. 2024 Jun 8;7:705. doi: 10.1038/s42003-024-06394-0 (PMC11162441; doi:10.1038/s42003-024-06394-0)
Supplement: Supplementary file 3 — Description of Additional Supplementary Materials [file 42003_2024_6394_MOESM3_ESM.docx]

**Description of Additional Supplementary Files**

**File name:** Supplementary Data

**Description:** The source data behind the graphs in the paper

**File name:** Supplementary Movie 1

**Description:** Comparative [Ca2+] imaging. (AVI, 17.8 MB)

**File name:** Supplementary Movie 2

**Description:** Functional highlighting of actively signaling cells by CaMPARInano. (AVI. 19.3 MB)

**File name:** Supplementary Movie 3

**Description:** Triple-function imaging of Ca2+, cGMP, and cAMP. (AVI. 4.1 MB)
